# Self-management of type 2 diabetes mellitus: a qualitative investigation from the perspective of participants in a nurse-led, shared-care programme in the Netherlands

**DOI:** 10.1186/1471-2458-8-91

**Published:** 2008-03-18

**Authors:** Albine Moser, Harry van der Bruggen, Guy Widdershoven, Cor Spreeuwenberg

**Affiliations:** 1 Department of Health Ethics and Philosophy, Care and Public Health Research Institute, Maastricht University, The Netherlands; 2 Department of Nursing Science, Care and Public Health Research Institute, Maastricht University, The Netherlands; 3 Department of Health Care Studies, Care and Public Health Research Institute, Maastricht University, The Netherlands

## Abstract

**Background:**

Diabetes mellitus is a major public health problem. Little is known about how people with type 2 diabetes experience self-management in a nurse-led, shared-care programme. The purpose of this article is to report an empirically grounded conceptualization of self-management in the context of autonomy of people with type 2 diabetes.

**Methods:**

This study has a qualitative descriptive, and exploratory design with an inductive approach. Data were collected by means of in-depth interviews. The sample consisted of older adults with type 2 diabetes in a nurse-led, shared-care setting. The data analysis was completed by applying the constant comparative analysis as recommended in grounded theory.

**Results:**

People with type 2 diabetes use three kinds of self-management processes: daily, off-course, and preventive. The steps for daily self-management are adhering, adapting, and acting routinely. The steps for off-course self-management are becoming aware, reasoning, deciding, acting, and evaluating. The steps for preventive self-management are experiencing, learning, being cautious, and putting into practice. These processes are interwoven and recurring.

**Conclusion:**

Self-management consists of a complex and dynamic set of processes and it is deeply embedded in one's unique life situation. Support from diabetes specialist nurses and family caregivers is a necessity of self-managing diabetes.

## Background

Diabetes mellitus constitutes a serious public health problem. The prevalence of diabetes mellitus is increasing [1]. The total number of people with diabetes is expected to increase from 171 million in 2000 to 366 million in 2030 [2]. The most significant demographic change of diabetes prevalence world-wide appears to be the increase in the proportion of people older than 65 years [2]. In the Netherlands, type 2 diabetes accounts for about 90% of all diabetes cases [3].

Western countries should establish a health-care system that meets the needs of the population [4]. Shared-care is an approach to provide needs-based care for chronically ill people [5]. In the Netherlands, several types of shared-care models have been implemented [6] in order to treat people with diabetes. In the Maastricht shared-care model, diabetes patients have been allocated to three types of care pathways: (a) patients with complicated health problems that may require radical medical decisions are referred to a medical specialist; (b) general practitioners provide basic care for the less complex cases and home health-care nurses provide the essential daily diabetes care; and (c) the diabetes specialist nurse (DSNs) has an intermediate position between the medical specialist and the general practitioner. DSNs work independently with their patients and are responsible for the care of the ones whose health status is stable but complex [7]. These patients require medium-intensity care [8]. DSNs function in both hospitals and primary care settings. This nurse-led, shared-care programme consists of a diabetes outpatient department at Maastricht University Hospital and specialty clinics in general practices. DSNs follow the agreed guidelines and protocols [7]. The tasks of the DSNs are to provide direct patient care, to organize and coordinate care (including medical care) for individual patients, and to provide advice and health education to patients and other care providers [8]. The result is that this particular nurse-led, shared-care setting is characterized by a mix of specific medical care and advanced nursing practice [9].

One of the goals of nurse-led, shared-care is to encourage people with diabetes to be active participants in their own care [5,8]. A literature review of the patient's perception of autonomy showed that autonomy has empirically been studied in the hospital, the nursing home, and home health care, but not in this particular setting [10]. Specifically, people with type 2 diabetes are required to manage many aspects of diabetes themselves on a life-long basis. The responsibility for day-to-day disease management shifts from health-care professionals to the individual [11]. Self-management places a large burden on patients [12]. It is a complex task involving diet, skin care, medication and insulin administration, exercise and rest, self-monitoring, and consulting health-care professionals. This requires researchers to take the self-management of chronically ill people into consideration as a general experience in their lives [13].

Our study is part of an ongoing research project regarding the autonomy of people with type 2 diabetes in a nurse-led, shared-care setting [9,10,14]. The long-term purpose of this project is first, to identify what issues need particular attention to foster patient autonomy in diabetes care provided by DSNs and second, to formulate recommendations to promote patient autonomy on the individual as well as policy level. The design of the ongoing research project is qualitative explorative which focuses on patient autonomy as perceived by older adults with type 2 diabetes mellitus. In a previous article we report how people with type 2 diabetes who are being cared for by a DSN in a nurse-led, shared-care unit view autonomy [9]. The core category 'competency in shaping one's life' describes how people with diabetes exercise their autonomy. Competency is the individual repertoire of skills that includes recognizing the possibilities and having the abilities, capacities, and expertise that allow people to shape their own lives. This implies that people with diabetes initiate and complete various daily actions, which are, in fact, dimensions of autonomy. To shape one's life means that a person actively strives towards a form of autonomy that is exactly right for only this particular person. Autonomy is based on characteristics that are unique to this person, and it is flexible with regard to changing health conditions and life situations. Shaping one's life is a construct of joining various dimensions of autonomy. Thus, the combination of the dimensions of autonomy is not fixed, but rather a mix of what seems most appropriate at a given time. We found seven dimensions of autonomy: identification, self-management, welcomed paternalism, self-determination, shared decision-making, planned surveillance, and responsive relationships. Each of the seven dimensions gives a different outline of competency for shaping one's life. In this article we focus on the dimension self-management.

During the interviews and analysis, we became aware of the dynamic character of the seven dimensions of autonomy. Therefore, we investigated the processes that underlie these dimensions. Most of the dimensions are shaped by more than one process, and the analysis provided a vast amount of data. To do justice to our findings, we chose to classify them for presentation. According to Sandelowski [15], qualitative findings can be grouped along temporal, thematic, event, or subject lines. We have chosen to make a thematic cut since self-management is very important to people with diabetes. In this study we define self-management as encompassing activities pertaining to taking care of one's health and diabetes. Self-management includes skills for activities of daily living (regular foot care) and the instrumental activities of daily living necessary for the treatment regimen (preparing diet meals, contacting the nurse). Self-management also refers to decision-making. Skills for effective self-management activities and deciding on self-management issues are intertwined [9].

### Literature review

In relation to nursing, Orem [16] describes three phases of self-care: the investigative, judgmental, and decision-making phases. Price [17] reports a diabetes self-management model for adults with type 1 diabetes that has two phases. Phase one, which consists of getting regulated, has four stages: trying it out, figuring it out, trial and error, and basic routine. Phase two consists of being regulated, with two substages: basic routine and applies basic routine to new diabetic situations. Ellison and Rayman [18] describe the experience of self-management of women with type 2 diabetes, who are expert self-managers. They move through three phases: management-as-rules, management-as-work, and management-as-living. Paterson and Thorne [19] describe the development of self-management expertise of people with type 1 diabetes. The four phases are passive compliance, naïve experimentation, rebellion, and active control. Paterson and Thorne [20] report how people with type 1 diabetes who are experts make everyday self-care decisions regarding unanticipated blood glucose levels. The five components in both familiar and unfamiliar situations are assessment of risk, comparative analysis, diagnosis, choice of action, and evaluation. Thorne et al. [21] did a secondary data analysis of how persons with expertise in self-managing type 1 and type 2 diabetes, HIV/AIDS, and multiple sclerosis learn everyday self-management decision-making. The authors identify three phases: assuming control, fine-tuning, and evaluating.

Research has focused mainly on people with type 1 diabetes and women with type 2 diabetes who are expert self-managers. It is less well understood how the "average" older adult person with type 2 diabetes manages diabetes. Additionally, the literature generally focuses on the development of self-management expertise, while we start from the notion of patient autonomy. Therefore, we want to answer the research question: "What are the processes through which older adults self-manage their diabetes in the context of autonomy?". The purpose of this article is to report an empirically grounded conceptualization of self-management in the context of autonomy of people with type 2 diabetes. This research is primarily not about the sufficiency of self-management in terms of outcomes such as well-regulated glycaemia control instead our attention centres on the personal understanding of the self-management of people with diabetes. Nevertheless, medical issues such as good blood results are part of the patient's experience of self-management.

## Methods

The design of this study is qualitative descriptive and exploratory, with an inductive approach as described in grounded theory [22]. According to Strauss and Corbin [22], the grounded theory approach is used because little is known about autonomy and self-management of people with type 2 diabetes in a nurse-led, shared-care setting. This method starts from the experience of the group under study, not from theories derived from the literature which has the benefit that the findings fit with practice. Grounded theory provides a way to go beyond experience – to move it from description of what is happening to understanding the process by which it is happening [23].

### Participants

We carried out the study with type 2 diabetes patients who were enrolled at the Maastricht Shared-care Unit VII. Fifteen people with type 2 diabetes participated in this study. The 6 women and 9 men were aged 55 to 77 years. They had been diagnosed with type 2 diabetes 1 to 16 years earlier. Eleven participants had general secondary or intermediate vocational education, and 4 had university education or higher vocational education. Eight participants were insulin dependent, and the remaining 7 were not. On average, the duration of the treatment by DSNs was 2.4 years and the participants had visited the DSNs 8.7 times. We invited patients who had been enrolled for at least 1 year at the nurse-led, shared-care clinics in the Maastricht region to participate in the study. All respondents were Dutch. People with confirmed and stable type 2 diabetes but with a complex health status, were included. Patients who lived independently in the community and were able to complete an interview of about 1.5 hours were included too. Type 2 diabetes patients with cognitive impairment and severe geriatric symptoms (diagnosed by a physician), newly diagnosed patients, and those for whom the interview was too great a burden were excluded. Elderly type 1 diabetic patients and type 2 diabetic patients who lived in an assisted-housing environment such as nursing homes or homes for the elderly were also excluded. The researcher, in cooperation with DSNs and the project manager from the nurse-led, shared-care unit, selected the participants with the aid of theoretical sampling [22]. The sample of this study stems from a common parent sample which is the basis of the long-term project mentioned earlier. The analyzed population is the same as in previous publications [9,14]

### Data collection

Fifteen in-depth interviews took place from March to September 2003. All participants were interviewed at home. The interview guide consisted of open-ended questions, for example, What diabetes care activities do you carry out? What do you do when you have a problem caused by the diabetes? Interviews were tape-recorded and transcribed verbatim. Field notes were taken during and after each interview. One researcher (AM) conducted all interviews.

### Data analysis

We analysed the data by applying constant comparative analysis as recommended in grounded theory [22]. In this approach, theory arises inductively from the interview data and deductively through constant comparison. Inductive coding was used to examine the phenomenon under study. We provided initial open coding for each interview and the field notes. We divided the data into small pieces and grouped these codes in larger subcategories and categories. We compared and contrasted the subcategories and categories and formulated concepts. Theoretical coding was used to develop models that clarify the relationships among concepts. We put the concepts back together to find patterns and processes and derived theoretical constructs by clarifying the relationships among the concepts. After analysis of 12 interviews, conceptual saturation occurred. The remaining 3 interviews were used to confirm and verify the analysis and findings. During the whole analysis, from open coding to writing down the findings, we had a continuous dialogue with the data. Memos were taken and analysed in each step of the analysis and helped to formulate the findings.

### Ethical considerations

After receiving detailed information about the research, the participants gave written informed consent prior to the interviews. The respondents were assured that interview data would be dealt with confidentially. Anonymity was secured by code-numbering the interviews. The ethics commission of Maastricht University Hospital and Maastricht University gave ethical approval.

### Trustworthiness

We assured credibility [24] by using multiple methods of data collection such as in-depth interviews and field notes (methodological triangulation). Several investigators were part of the project groups, and two researchers were involved in the analysis and interpretation of data (investigator triangulation). Different sources of the same information, such as numerous interviews, were used to validate the findings (data triangulation). Several distinctive questions were asked regarding topics related to autonomy and self-management; participants were encouraged to support their statements with examples, the interviewer asked follow-up questions, and the researchers studied the data from raw interview material until the theory emerged to provide the scope of the phenomenon under study (prolonged engagement). The researchers concentrated on the aspects of the interviews and field notes that were most relevant to the issue under study and focused on them in detail; we read data, analyzed them, theorized with them, and revised the concepts accordingly until the findings provided depth (persistent observation). Throughout the inquiry, the project group held meetings to review and explore scientific and organizational aspects of the project (peer debriefing). We sought cases and events that disconfirm evolving categories until the theory accounted for all cases (negative case analysis). We guaranteed transferability [24] by providing descriptive data of the study context (thick description) on request to enable readers to evaluate whether the findings are transferable to other care contexts. Dependability and confirmability [24] were assured by an academic auditor. The auditor checked whether the analysis was in line with accepted standards and examined the analysis process and records for accuracy.

## Results

**Figure 1:**
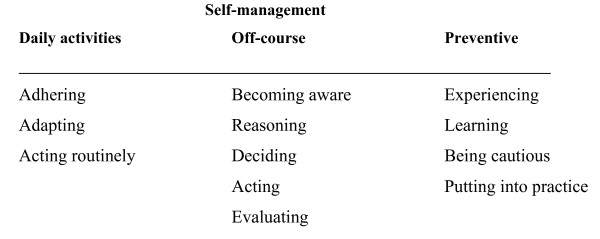
Self-management processes as described by people with type 2 diabetes: daily, off-course and preventive self-management.

One of the seven ways people with type 2 diabetes pursue their autonomy as 'competency in shaping one's life' is self-management. People with diabetes exercise self-management by a set of particular activities. We consider these specific series of practices as self-management processes. These processes provide understanding how self-management in relation to autonomy actually happens and is achieved. The analysis of participant's descriptions of their self-management practices as related to autonomy resulted in three processes: daily, off-course, and preventive self-management. Figure 1 gives an overview of the process steps. Most participants reported all three kinds of self-management. Some practised all of them while others reported (a) particular self-management process(es) at a given time. Additionally, some participants said that they took process steps in a linear order, while others reported that they occasionally repeated some steps. Some said that the processes were recurrent and cyclical. Some might not take all steps, and others might take their steps in a slightly different sequence. In this study, the participants described short-term (off-course) and long-term (daily and preventive) self-management processes. They also mentioned that a short-term self-management strategy might turn into a long-term one. People evaluate the effect of the short-term decision made and the action taken. The decision to turn a short-term self-management strategy to a long-term one depends upon how well the off-course event was dealt with. We report analytical steps, but in practice these steps occur simultaneously and in an interactive process. 

### Daily self-management

Daily self-management is related to daily diabetes activities such as administering insulin, exercising, and resting. It includes activities that belong to the treatment regimen and activities for staying healthy in general. Daily self-management consists of three steps: adhering, adapting, and performing routinely.

#### Adhering

People with diabetes adhere with the treatment regimen as prescribed by healthcare professionals. They stick to the rules and carry out self-management activities as required by the treatment plan, e.g., they take their medication exactly on time.

"At the very beginning, I did as I was told. I started taking my medication and eating precisely on time, doing exercises and losing weight, going to have my blood and blood pressure checked every 3 months."

Some participants said that they adhered rigorously to 'new' rules when they had to deal with a new treatment. They had been suffering from diabetes for a longer period and already had some experience with diabetes self-management. This kind of adherence is temporary until people with diabetes become more confident with the new treatment.

"The units of insulin, yes, I really need supervision and monitoring. I do not change one unit without consulting my nurse. I am still somehow inexperienced at using insulin. I know my body very well, but I do not know its reaction (to insulin) yet. (...) At the beginning, I asked a lot of questions. Now I have gained more confidence, and I ask fewer questions."

#### Adapting

People with diabetes adapt to the prescribed treatment regimen. They make small adaptations, like changing their food patterns. Then they alter the treatment to fit their lifestyles better, or because they perceive their measures as good enough, e.g., acceptance of a fairly low blood sugar level. Adapting the treatment is often related to self-management issues that people are familiar with. They deal with self-management strategies more flexibly.

"My average blood sugar level has been decreasing in the last few months. At my last visit it was low. I am on oral medication and I stuck to a rigid diet. I handle my diet more flexibly now. Nowadays I use more noodles and rice in my daily cooking."

#### Acting routinely

Diabetes care activities become a habit. People with diabetes have routines that are embedded in their personal lives. They are confident in these activities.

"I do the same thing every day. It has become a habit. In the morning, I take my medication, I take care that I eat a mid-morning snack, then I cook. I rest a little. Later in the afternoon, I go for a walk or take some other exercise. I take a piece of fruit with me. I have dinner. I do a self monitoring sugar profile once every 2 weeks."

### Off-course self-management

Off-course events include certain health problems caused by diabetes that may occur – sometimes repeatedly, e.g., unanticipated blood sugar levels. When their blood sugar concentrations are below or above a certain value, people take action in response. Off-course self-management includes five steps: becoming aware, reasoning, deciding, acting, and evaluating.

#### Becoming aware

People recognize certain patterns that are unusual. They become aware of irregularities by listening to their bodies, by learning from previous experience, and by monitoring their glucose parameters. Awareness can be improved with a diabetes education course and by consulting the DSNs.

"I feel it when my sugar level is not what it should be. I feel dizzy and sometimes I become irritated. It happens when I work hard; for example, when I clean the house."

#### Reasoning

People try to reason why irregularities occur. They explore their self-management activities critically to find an explanation for their unusual blood sugar levels. People with diabetes also consider other bodily symptoms and glucose parameters to support or refute possible causes.

"I checked my glucose levels yesterday. The glucose profile for the day was quite high. I had a very busy weekend, and a lot of friends visited us. I also ate different food. I really had peaks. I think it happened because of the busy weekend."

#### Deciding

People with diabetes make decisions to solve certain off-course events. They decide how to deal with the health problem at hand.

"Some time ago I had hypos in the evenings. I decided to decrease the amount of the fast-acting insulin."

#### Acting

People with type 2 diabetes take action on the relevant decision. They actively undertake specific actions to resolve the off-course event, e.g., by taking some extra fast-acting carbohydrates.

"When I notice that my sugar level is too low, I eat some chocolate."

#### Evaluating

After carrying out a certain self-management activity, people observe the reactions of their bodies and their blood sugar parameters. They critically evaluate the effect of their decisions and actions.

"Some time ago, I had hypos 3 days in a row. Then I decreased the amount of insulin that I injected in the morning. And it worked out well. I have not had hypos for quite some time."

### Preventive self-management

Preventive self-management means that people do certain things to prevent health problems. Diabetes-related health problems encompass short-term complications, such as hypoglycaemia, and secondary long-term complications, such as blindness. Preventive self-management is composed of four steps: experiencing, learning, being cautious, and putting into practice.

#### Experiencing

People may undergo a health experience such as a severe hypoglycaemia. This experience is perceived as very unpleasant, frightening, or threatening.

"I have had some hypos (hypoglycaemia) during my vacation. I was not eating the right things. I did not eat carbohydrates because I wanted to loose weight. At the same time, I walked a lot and then I had several hypos. It was very unpleasant."

People are sometimes faced with negative health events in the family. In one case, a family member had diabetes and suffered from severe health problems, some of which were short-term and some long-term.

"I am afraid of getting a really bad hypo. My mother had a very severe hypo. She was unconscious, and I thought that she was dead. I am being very careful not to get one, but I'm still afraid. I am being very careful to prevent anything like that."

#### Learning

People learn about short-term and long-term complications caused by diabetes. They gain practical knowledge from experience with them. The DSNs provide a lot of technical and generic learning. People also get information by reading diabetes literature and taking part in diabetes education programs.

"The nurse explained to me that I can get a lot of health problems if I do not take care of my diabetes. I can get problems with my feet, kidneys, and eyes. It is important to know all these things."

#### Being cautious

People with type 2 diabetes are cautious in situations that could cause short-term and long-term health problems. They also listen to their bodies to recognize certain symptoms early.

"The nurse explained the symptoms of hyper- and hypoglycaemia to me. She also gave me some literature about it. I am much more cautious about these things now and I observe and listen more carefully to my body."

Furthermore, people with diabetes monitor their blood sugar levels regularly. They are cautious because such irregularities might cause long-term problems.

"A few days ago, my sugar levels for the day were very unstable. I need to keep them within a certain range because of all the health problems that might develop over the years."

#### Putting into practice

People with diabetes put particular strategies into practice to prevent a short-term health complication such as hypocalcaemia.

"If I go cycling, I always have an apple in my bag to get enough carbohydrates."

People are aware of the long-term health problems of diabetes. They implement courses of actions to prevent these complications.

"I try to slow down the process of long-term complications. I cycle a lot. It helps keep my sugar levels low and stable. I cycle, I take long walks, and I keep myself busy."

### The involvement of diabetes specialist nurses and family caregivers

#### Diabetes specialist nurses

DSNs are important to diabetes self-management. They teach, guide, and advise people with diabetes about self-management issues. DSNs confirm, modify, monitor, and change self-management strategies. They help to develop self-management schemes and skills.

"Now I know that I will see her regularly. If something is not right, she explains to me what went wrong and what I need to change. She monitors my health status. She advises me. If I need to know something, I give her a call."

The DSNs are available as a safety net to assist people when they have run out of self-management strategies or find themselves in unfamiliar situations.

"Some time ago, I had a hypo during the night. I did not know how to interpret this. It makes me feel insecure. I called my nurse."

The role of the DSNs depends on the complexity of the self-management. If the complexity decreases or the diabetes management becomes stable, the role of DSNs may become less significant. In some cases, as with radical changes in the treatment plan or episodes of severe illness, the role of DSNs may expand temporarily.

"I think that, if I have serious health problems, her (the nurse's) role will get bigger. She needs to take over the lead. This is not necessary right now because my condition is stable. I handle things myself."

#### Family caregivers

Diabetes self-management becomes part of the family life. People with diabetes embed the diabetes in the family context in different ways. All participants in this study state that family caregivers support them in their diabetes self-management. The involvement differs from person to person. Some participants call on their relatives in an emergency.

"He (the participant's husband) knows how to inject insulin. We learned it together. He also pricked my finger with a needle. I do everything myself now, and I try to solve all my own problems, but he takes care of me too. He only comments on my diabetes things every now and then, but he needs to know everything. This is important to me. If I am unconscious and need to be admitted to the hospital, he must be able to provide the necessary information."

Some family caregivers help the person with some self-management activities, for example, by preparing meals.

"My wife takes care of the cooking. I took a course about diet, and my wife knows how to cook for someone with diabetes. She takes care of my meals and my eating pattern. I manage everything else myself."

Some participants see the family caregiver as a partner or companion with whom they share all self-management. In these cases, family caregivers have a significant part in every single self-management step.

"She' (the participant's wife) 'is my assistant. She cooks, she thinks with me, she takes care of the medication. I also do all these things, but she keeps me company in managing the whole diabetes thing, which is essential to me."

## Discussion

The purpose of this article is to report of an empirically grounded conceptualization of self-management in the context of autonomy of people with type 2 diabetes.

Diabetes self-management is a lifelong matter and is clearly directed towards care. This study shows that self-management, as described by people with diabetes themselves, goes far beyond compliance and 'good blood control'. It takes shape within the context of a unique life in which one establishes one's personal self-management strategies. Not all the participants attained or maintained the same level of self-management [17]. For some, self-management relates to daily activities based on a firm structure. Others have developed sophisticated self-management skills that allow situational modifications. Furthermore, most participants said that they did not manage diabetes equally well every day. This is congruent with other research [18,19,21]. As Ellison and Rayman [18] put it, "100% some days and 40% other days".

The processes of self-management require a mix of cognitive (e.g. reasoning), practical (e.g. acting), and social (e.g. communicating) skills. In line with our findings, empirical researchers report an interplay of cognitive and practical skills in their descriptions of self-management processes [17-21]. In this study, the importance of social skills comes to the fore because people request social support in self-management. The data suggest that social support from DSNs and family caregivers is essential and fosters self-management. Research mainly reports supportive relationships with nurses [17-21], but rarely mentions the relationships with family caregivers and their role in self-management. The participants in our study did not refer to support from peers [20,21], but some offered their services voluntarily to diabetes support groups.

In previous research, the participants were recruited by means of a diabetes newsletter [17], a diabetes teaching and research centre [18], self-nomination or nomination by an internist [19,20], and nomination by primary care specialists [21]. Apparently, the participants were not treated by nurses. In this study, DSNs are the main caregivers. Their tasks include both medical and nursing procedures. Our findings confirm characteristics that seem common across studies but highlight aspects that are specific to nursing, such as preventive diabetes care. Preventive self-management has not yet been conceptualized in the literature. In public health nursing, it is critical to avoid both short-term and long-term complications. Perhaps we have been led to turn our attention to preventive self-management because of the long-term and care nature of nurse-led, shared-care and our participants are older adults. Many of them suffer from more than one chronic condition, and diabetes-related complications might be linked to functional losses and increased risk of care dependency. They associate preventive self-management with sustaining freedom and prolonging independence. Most of our participants had been confronted with the complications of family members or had developed some of their own. Such experience influenced their own self-management. Attempting to avoid severe complications seems to be the motivator of preventive self-management.

Self-management is heavily influenced by professional recommendations. Understanding the processes that underlie self-management will enable nurses to provide diabetes counselling, which goes beyond generic technical education, symptom management, compliance, and metabolic control. Nurses should focus on providing people with diabetes with the necessary cognitive, social, and practical skills needed for autonomously exercising of self-management. Continuous social support is essential for self-management. DSNs are in a position to provide long-term counselling for people with diabetes and, in order to do so, they should build collaborative and accommodating relationships with their patients, and if appropriate, with family caregivers. Especially in the care for older adults DSNs should focus on preventive self-management. In some cases, DSNs need to provide support to people with diabetes and to family members which are (sometimes closely) involved in the care for the person with diabetes. In these cases DSNs should include family caregivers in their counselling. However, DSNs should keep autonomous self-management as desired by the person with diabetes as the focal point of their counselling.

People with diabetes frequently mention that their family members assist them with diabetes-specific self-management activities. In our study, people with diabetes experience the participation of close ones as positive, thus enhancing autonomous self-management. Family members should support the person with diabetes by being attentive to the self-management wishes and needs of their close ones. The involvement of family caregivers has a different meaning to each individual person with diabetes and there are various ways family members can participate in diabetes self-management. Family caregivers should take part in the consultancies with the DSN if the person with diabetes wishes so. In addition, family members too need DSNs to find answers to their caring questions to contribute effectively to the diabetes self-management of their kin. We encourage family members to assist their kin with diabetes to a degree that they view themselves as self-managers and are able to maintain an equilibrium between self-management autonomy and reliance on close ones.

A limitation of this study is that the interviews took place within a restricted geographical area. Diversity in sampling ensured data that covered a range of behaviour in different situations. An interview requires certain physical and cognitive strengths; therefore, people with severe health problems were not interviewed. Another limitation is the fact that nurses who work at the nurse-led, shared-care unit selected the participants. At the time of the study, all participants were treated by DSNs. We could not interview people who were enrolled at the nurse-led, shared-care unit but did not comply with the follow-up regime. The findings might not be applicable to the so-called non-compliers. These people might associate non-compliance with a need for autonomy or see it as a way of asserting independence. The results are comparable to those programmes similar to the one described here. First, the scope and tasks of nurses should be comparable to those of advanced practice nurses at the general practitioner's or health visitors. Secondly, the context in which care is given should have common features such as the ones in follow-up monitoring care settings. Third, the patient population should be similar to our sample of people with diabetes without acute and serious health problems.

## Conclusion

Autonomy for people with diabetes requires competency in shaping one's life. Competency in shaping one's life means that individual autonomy is achieved by matching up various dimensions of autonomy. Self-management is an important dimension in realizing autonomy and consists of daily, off-course, and preventive self-management. These processes are interwoven and recurring. Complex processes shape the three kinds of self-management because it is much more than learning and complying with a treatment regimen. Self-management is deeply embedded in one's unique life situation. Support from DSNs and family caregivers are a prerequisite for people to self-manage the diabetes.

## Abbreviations

DSNs: Diabetes specialist nurses

## Competing interests

The author(s) declare that they have no competing interests.

## Authors' contributions

AM contributed to the conception and design, acquisition, analysis and interpretation of data and drafting of the manuscript. HvdB participated in the analysis and interpretations of the data and made substantial contributions to the manuscript. GW and CS were involved in the conception and design, and reviewed the manuscript. All authors read and approved the final manuscript.

## Pre-publication history

The pre-publication history for this paper can be accessed here:


